# Concentration‐ and time‐dependent behavioural effects of ethanol on *Lumbriculus variegatus*

**DOI:** 10.1111/gbb.70006

**Published:** 2024-10-15

**Authors:** Aidan Seeley, Romessa Mahmood, Caitlin Bellamy, Elis G. Roome, Benjamin S. Williams, Nia A. Davies, Melisa J. Wallace

**Affiliations:** ^1^ Swansea Worm Integrative Research Laboratory (SWIRL) Swansea University Medical School, Swansea University Swansea Wales UK

**Keywords:** behaviour, ethanol, invertebrate, *Lumbriculus variegatus*, tolerance

## Abstract

Ethanol is one of the most widely used drugs in the world. Ethanol induces profound physiological and behavioural responses in invertebrate model organisms, such as *Caenorhabditis elegans* and *Drosophila melanogaster*. *Lumbriculus variegatus* (Annelida, Oligochaete) is an aquatic worm which shows behavioural responses to common drugs and thus is potentially useful in pharmacological research. The effects of ethanol are unknown in this organism. In this study, we examine the effects of acute exposure to ethanol (0–500 mM) on the stereotypical movements and locomotor activity of *L. variegatus* and examine the concentration‐ (0–500 mM) and time‐dependent (0–210 min) effects of ethanol in *L. variegatus*. We show that ≥250 mM ethanol reversibly reduced the ability of tactile stimulation to elicit stereotypical movements, namely body reversal and helical swimming and locomotor activity (*p* < 0.05, *N* = 8). We also found that 2 min of exposure to ≥250 mM ethanol rapidly induces steady‐state hypokinesis (*p* < 0.05, *N* = 11) and confirm ethanol absorption into *L. variegatus* tissues. Additionally, we also observed acute ethanol tolerance after 150 min of exposure to 500 mM ethanol (*p* < 0.05, *N* = 24). This study is the first to report the behavioural effects of ethanol in *L. variegatus*. Our results show that this is a model organism for use in ethanol studies, providing further evidence for its utility in pharmacological research.

## Graphical abstract

Ethanol‐treated *Lumbriculus variegatus* exhibit decreased responses to tactile stimulation and locomotor activity after 10 min exposure, while acute ethanol tolerance emerged after ≥150 min. Created with BioRender.com.

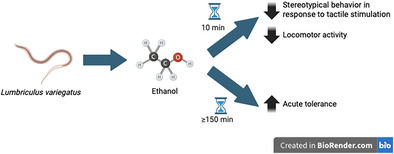

## 1 INTRODUCTION

Ethanol is one of the most widely used drugs in the world.^1^ Ethanol produces concentration‐ and time‐dependent effects on locomotion and behaviour of invertebrate model organisms such as *Caenorhabditis elegans* and *Drosophila melanogaster*, with effects comparable to those of higher organisms.^2^, ^3^, ^4^ While ethanol does not exert its pharmacological effect through one specific receptor, many partial mechanisms of action have been proposed, for example, inhibition of glutamatergic signalling,^5^, ^6^ potentiation of cAMP signalling^7^ modulation of neuropeptide‐Y related pathways,^8^, ^9^ GABAergic synaptic inhibition^10^ and membrane perturbation.^11^

Acute ethanol exposure in *C*. *elegans* has been shown to decrease locomotion and pharyngeal pumping^3^, ^12^ with similar effects observed in *Drosophila*.^9^, ^13^ Low‐dose exposure to ethanol (17–52 mM) produces hyperactivity in *C*. *elegans* while concentrations ≥100 mM decrease motility.^3^, ^14^, ^15^, ^16^ These low doses of ethanol are physiologically relevant to human studies, with 0.1% blood alcohol (corresponding to 21.7 mM ethanol) being a common legal driving limit.^2^ Like vertebrates such as humans, rats and mice, *C*. *elegans* and *Drosophila* develop tolerance to ethanol.^4^, ^8^ Ethanol tolerance is a reduction in the intensity of the effects of ethanol upon continuous or repeated exposure and can be divided into three forms; acute, rapid and chronic. Acute, or in session, tolerance occurs *during* exposure to ethanol; rapid tolerance is expressed after a single exposure and subsequent metabolism of ethanol; while chronic tolerance occurs after repeated ethanol exposures.^17^, ^18^, ^19^, ^20^ All three types of tolerance have been observed in *C*. *elegans* and *Drosophila*.^4^, ^8^, ^14^, ^21^, ^22^, ^23^

*L*. *variegatus* is an aquatic, regenerative, asexually reproducing annelid worm inhabiting shallow freshwater ponds, lakes and marshes^24^ which has been used as an ecological indicator species^25^, ^26^, ^27^, ^28^, ^29^, ^30^ but is increasingly being used to study pharmacologically active compounds.^31^, ^32^, ^33^, ^34^ These studies have utilised behavioural parameters such as locomotor activity, stereotyped behaviours of body reversal and helical swimming, feeding and reproduction as experimental endpoints. *L*. *variegatus* represented a novel invertebrate organism for whole organism in vivo research that presents some advantages over other invertebrate organisms, such as the larger size of *L*. *variegatus* (50–80 mm) compared with *C*. *elegans* (~1 mm) which allows for evaluation of individual organism effects when exposed to pharmacological compounds without the requirement for microscopy equipment or use of multiple organisms per test condition.^34^ Moreover, the liquid culture in which *L*. *variegatus* are exposed to compounds allows for improved accuracy of drug exposure, as use of solid nematode growth medium plates, often used in studies of *C*. *elegans*, is often an underestimate and not directly experimentally comparable to the same concentration in a liquid medium.^35^

In the present study, we characterise the behavioural effects of acute ethanol exposure using methodology analogous to studies observing concentration‐dependent effects of ethanol in *C*. *elegans*.^3^ We show, for the first time, the effects of concentrations of ethanol that produces intoxication in humans^12^ and ethanol concentrations previously used in studies of *C. elegans* ^3^ within *L. variegatus*. We present the effects of ethanol exposure on the effect of tactile stimulation to evoke stereotypical behaviours^24^ and locomotor activity.^34^ For the first time, we describe concentration and time‐dependent effects in response to acute ethanol exposure and ethanol absorption in the novel *Lumbriculus variegatus* model. Furthermore, we also show that, as in *C. elegans* and *Drosophila*, ethanol exposure can produce acute tolerance in *L. variegatus*.^4^, ^8^, ^15^

## 2 MATERIALS AND METHODS

### 2.1 *L*. *variegatus* culture

*L. variegatus* were purchased from Alfa Fish Foods and laboratory‐reared in artificial pond composed of 1 mM NaCl; 13 μM KCl, 4 μM Ca(NO_3_)·4H_2_O; 17 μM Mg(SO_4_)·7 H_2_O; 71 μM HEPES buffer in UV‐treated deionised water produced by Elix® Essential 3 UV Water Purification System.^34^ Cultured were maintained at room temperature (18–21°C), subject to a 16:8‐h light–dark cycle and fed TetraMin flakes and 10 mg/L spirulina weekly. Artificial pond water was subject to continuous aeration and water filtration using commercial air stones and aquarium filters, respectively. The pH was not monitored or adjusted once the worms were placed in the water.^34^ Populations were maintained for a minimum of 3 months before experimentation to limit colony variation and to allow for population growth by asexual reproduction.^27^, ^34^ Prior to testing, individual worms were randomly selected, lacked any obvious morphological defects and ranged from 2 to 8 cm in length as per previous studies.^27^, ^31^, ^34^

### 2.2 Materials

Ethanol (≥99.8%) was obtained from Fisher Scientific (Leicestershire, United Kingdom) and diluted in artificial pond water. Artificial pond water was used as a vehicle control.

### 2.3 Ethanol effects on *L. variegatus* stereotypical movement

Experiments were conducted as previously described by Seeley et al.^34^ Briefly, 18–24 h before experimentation, individual *L. variegatus* was placed in each well of a Cellstar® 6‐well plate (Greiner Bio‐One) containing 4 mL of artificial pond water and kept at room temperature, subject to a 16:8‐h light–dark. After this acclimation period, the pond water was replaced and the baseline ability of the worm to respond to tactile stimulation was tested using with a 20–200 μL plastic pipette tip, alternately stimulating the anterior or posterior of the body. The artificial pond water was then removed and immediately replaced with the vehicle control (artificial pond water only) or ethanol (0–25 mM or 0–500 mM). After a 10‐min incubation, the worms were tested again using the same procedure. Solutions were aspirated from the well, and to remove any latent ethanol, fresh pond water was added, immediately aspirated and replaced with fresh artificial pond water. *L. variegatus* were then retested 10 min (10 min recovery) and 24 h (24 h recovery) after incubation in artificial pond water. Data were expressed as a ratio of the movement score while in treatment relative to baseline.

### 2.4 Ethanol effects on *L. variegatus* locomotor activity

Experiments were conducted as previously described by Seeley et al.,^34^ with the acclimatisation period the same as outlined above. Following this acclimation period, artificial pond water was replaced with 2 mL fresh artificial pond water to limit movement in the *z*‐axis, and baseline locomotor activity was recorded by rapid, sequential image collection with a 13‐megapixel camera at a rate of one image per second for 50 s. Images were then collected 10 min after removal and immediate replacement of artificial pondwater with the vehicle control (artificial pond water only) or ethanol (0–25 mM or 0–500 mM). Solutions were then removed, the wells washed and fresh artificial pond water added. Images were taken after 10 min (10 min recovery) and 24 h (24 h recovery) in artificial pond water.

Collected images were then analysed using ImageJ software by superimposing images taken each time point and using an area of known distance within each image to calibrate ImageJ to pixels per centimetre within each superimposed image set. To determine the area traversed by each worm, the foreground and background were separated using the thresholding functionality of ImageJ to separate the pixels activated by *L*. *variegatus* from those activated by the 6‐well plate. The total area covered by the *L*. *variegatus* before ethanol exposure, during ethanol exposure and both recovery timepoints was then determined based on the calibration of pixels/cm within ImageJ. Data were expressed as a percentage of the locomotor activity by *L*. *variegatus* compared with baseline conditions.

### 2.5 Onset of ethanol action

18–24 h before experimentation, one *L. variegatus* worm was placed in each well of a Cellstar® 6‐well plate (Greiner Bio‐One) containing artificial pond water only. Plates were subject to a 16:8‐h light–dark cycle and kept at room temperature. After this acclimation period, artificial pond water was aspirated and replaced with fresh artificial pond water. Locomotor activity before drug treatment (0 min) was recorded by rapid, sequential image collection using a 13‐megapixel camera at a rate of one image per second for 50 s. Image collection was repeated at 2‐min intervals following the removal of artificial pond water and immediately replacing artificial pond water with ethanol (0–500 mM) or vehicle control. Images were analysed as previously described^34^ with data expressed as a percentage of the locomotor activity covered by *L. variegatus* at 0 min.

### 2.6 Ethanol absorption

Ethanol absorption was measured using a commercial colourimetric assay (MAK076, Sigma‐Aldrich, Dorset, United Kingdom). In this assay, ethanol absorption was detected by a coupled enzyme reaction, which results in a colourimetric (570 nm) product, proportional to the ethanol present. Briefly, 18–24 h before experimentation, five *L. variegatus* per condition were added to a CellStar® 6‐well plate (Greiner Bio‐One). *L. variegatus* were exposed to artificial pond water or 500 mM ethanol and transferred to a 1.5 mL Eppendorf tube before being briefly washed with 500 μL of ice‐cold artificial pond water twice. Following washing, *L. variegatus* were homogenised using a Cole‐Parmer® motorised pestle mixer in 50 μL of ice‐cold artificial pond water. Homogenates were centrifuged at 16.1 RCF for 15 min and combined with Master Reaction Mix, the solution was mixed well using a horizontal shaker and incubated for 60 min at room temperature, and the absorbance was measured at 570 nm using a FLUOstar Omega microplate reader (BMG Labtech). The absorbances obtained were from three experimental repeats run in duplicate. Reported values are displayed per mg of protein, or relative to 10 min of exposure to 500 mM ethanol, with total protein content of *L. variegatus* homogenates quantified following Bradford's method^36^ using bovine serum albumin as the standard for quantification.

### 2.7 Acute tolerance to ethanol

18–24 h before experimentation, one *L. variegatus* worm was placed in each well of a Cellstar® 6‐well plate (Greiner Bio‐One) containing artificial pond water only, acclimatised and untreated baseline movement recorded before exposure (0 min) as described above. *L. variegatus* locomotor activity was then recorded after 10 min of exposure to 500 mM ethanol and at 20‐min intervals up to 210 min of ethanol exposure.

### 2.8 Statistical analysis

Statistical analysis was performed in GraphPad Prism 10 with *p* < 0.05 as the threshold for statistical significance.

## 3 RESULTS

### 3.1 Behavioural response to ethanol exposure

First, we sought to determine if *L. variegatus* would display behavioural effects when exposed to ethanol concentrations equivalent to physiologically relevant concentrations in humans by examining the effects of tactile stimulation to elicit *L. variegatus* stereotypical movements, and effects on locomotory activity.

**FIGURE 1:**
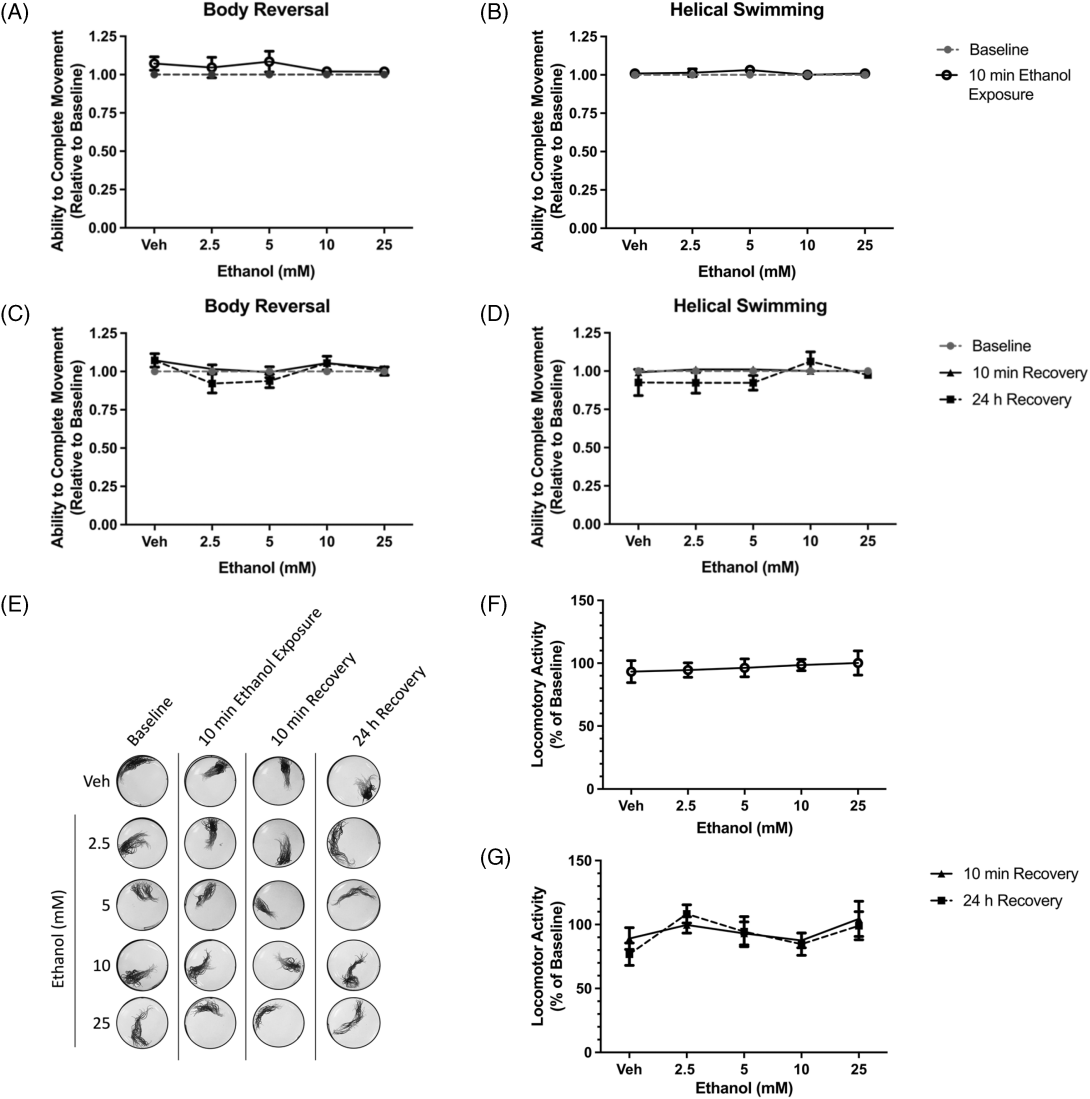
The effect of 10 min exposure to 0–25 mM ethanol on *Lumbriculus variegatus* behaviour. *L. variegatus* were exposed to ethanol (0–25 mM) for 10 min and tested for the ability of tactile stimulation to elicit (A) body reversal or (B) helical swimming. Ethanol was then removed and the ability of *L. variegatus* to perform (C) body reversal or (D) helical swimming was tested after 10 min and 24 h. Data were expressed as a ratio of the movement score after exposure relative to the movement score at baseline. (E) Representative superimposed images analysed in ImageJ showing the effect of 10 min of exposure to ethanol on locomotor activity measured before ethanol exposure (baseline), after 10 min of exposure to 0–25 mM ethanol (10 min ethanol exposure), 10 min after ethanol removal (10 min recovery) and 24 h after ethanol removal (24 h recovery). Quantification of the area covered by *L. variegatus* following (F) 10 min of exposure to 0–25 mM ethanol and (G) removal of ethanol for 10 min and 24 h are expressed as a percentage of the area covered at baseline. Analyses were conducted by comparing ethanol exposure conditions to baseline conditions by paired non‐parametric two‐tailed *t*‐test for stereotypical movement assays and paired parametric two‐tailed *t*‐test for locomotor activity. A two‐way ANOVA with Dunnett's post‐test was used to analyse 10‐min and 24‐h recovery time points compared with baseline conditions for *L. variegatus*. Error bars represent the standard error of the mean, *N* = 8 for each concentration. Veh: Artificial pond water only.

We observed that acute 10‐min exposure to low‐dose ethanol concentrations (0–25 mM) did not significantly affect the ability of *L. variegatus* to perform body reversal or helical swimming movements in response to tactile stimulation (*p* > 0.05, Figure 1A,B, *N* = 8). No effects on stereotypical movements were observed 24 h after exposure to ethanol (*p* > 0.05, Figure 1C,D, *N* = 8).

Locomotor activity of *L. variegatus* was similarly unaffected when exposed to ≤25 mM ethanol (*p* > 0.05, Figure 1F, *N* = 8). Moreover, 24 h after ethanol removal, locomotor activity was indistinguishable from pre‐exposure locomotion (*p* > 0.05, Figure 1G, *N* = 8).

**FIGURE 2:**
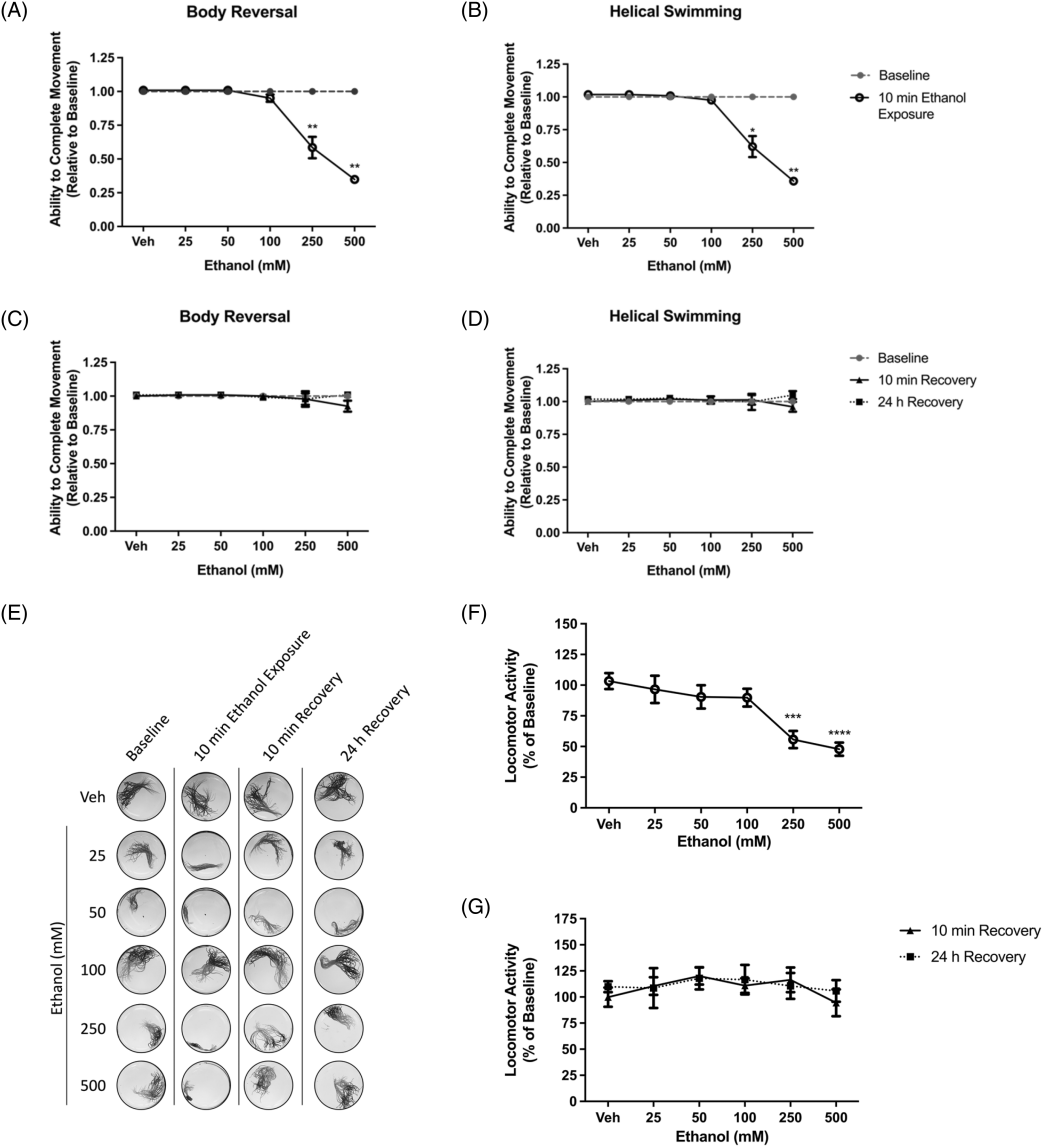
The effect of 10 min exposure to 0–500 mM ethanol on *Lumbriculus variegatus* behaviour. *L. variegatus* were exposed to ethanol (0–500 mM) for 10 min and tested for the ability of tactile stimulation to elicit (A) body reversal or (B) helical swimming. Ethanol was then removed and the ability of *L. variegatus* to perform (C) body reversal or (D) helical swimming was tested after 10 min and 24 h. Data were expressed as a ratio of the movement score after exposure relative to the movement score at baseline. (E) Representative superimposed images analysed in ImageJ showing the effect of 10 min of exposure to ethanol on locomotor activity measured before ethanol exposure (baseline), after 10 min of exposure to 0–500 mM ethanol (10 min ethanol exposure), 10 min after ethanol removal (10 min recovery) and 24 h after ethanol removal (24 h recovery). Quantification of the area covered by *L. variegatus* following (F) 10 min of exposure to 0–500 mM ethanol and (G) removal of ethanol for 10 min and 24 h are expressed as a percentage of the area covered at baseline. Analyses were conducted by comparing ethanol exposure conditions to baseline conditions by paired non‐parametric two‐tailed *t*‐test for stereotypical movement assays and paired parametric two‐tailed *t*‐test for locomotor activity. A two‐way ANOVA with Dunnett's post‐test was used to analyse 10‐min and 24‐h recovery time points compared with baseline conditions for *L. variegatus*. * *p* < 0.05, ** *p* < 0.01, *** *p* < 0.001, **** *p* < 0.0001. Error bars represent the standard error of the mean, *N* = 8 for each concentration. Veh: Artificial pond water only.

Having observed that to ≤25 mM ethanol did not have significant effects on *L. variegatus*, we sought to determine if higher concentrations of ethanol commonly used in studies of *C. elegans* would have an effect on this species. At higher concentrations of ethanol (25–500 mM), acute 10‐min exposure resulted in the reduced ability of tactile stimulation to elicit stereotypical movements at 250 and 500 mM (*p* < 0.05, Figure 2A,B, *N* = 8). The observed effects were reversible when ethanol was removed and replaced with artificial pond water for 10 min, with tactile stimulation eliciting stereotypical movements at a level indistinguishable from baseline conditions (*p* > 0.05, Figure 2C,D, *N* = 8). Additionally, no effects of acute ethanol exposure were observed on the ability of tactile stimulation to reduce stereotypical movement 24 h after exposure (*p* > 0.05, Figure 2C,D, *N* = 8).

*L. variegatus* locomotor activity was significantly reduced to 53.73 ± 7.05% (*t*_7_ = 6.28, *p* = 0.0004, *N* = 8) and 47.76 ± 5.42% (*t*_7_ = 9.46, *p* < 0.0001, *N* = 8) after exposure to 250 mM and 500 mM ethanol, respectively, compared with untreated, baseline conditions (Figure 2F). Ten min after the removal of ethanol, *L. variegatus* locomotor activity returned to pre‐exposure levels (*p* > 0.05, Figure 2G, *N* = 8).

### 3.2 Onset of ethanol effects

**FIGURE 3:**
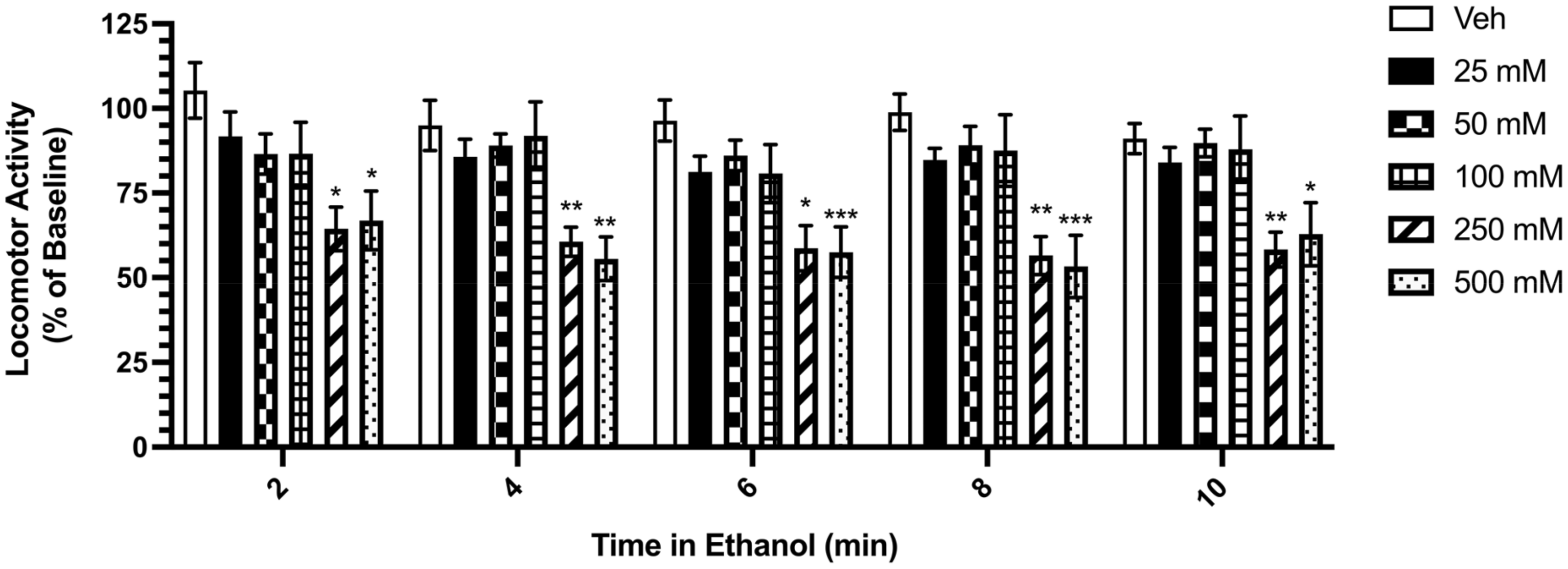
The onset of concentration‐dependent inhibitory effects of ethanol on *Lumbriculus variegatus* locomotor activity. *L. variegatus* were exposed to ethanol (0–500 mM) for 10 min and locomotor activity was measured before ethanol exposure and every 2 min for 10 min of exposure to 0–500 mM ethanol. Data were expressed as a percentage of the area covered before exposure (0 min). Analyses were conducted using a one‐way ANOVA with Dunnett's post‐test comparing ethanol‐treated *L. variegatus* to untreated controls (0 min). Steady‐state effects of ethanol exposure on *L. variegatus* locomotion were compared over time using a paired parametric two‐tailed *t*‐test compared with effects after 2 min of exposure to ethanol.*p < 0.05, **p < 0.01, ***p < 0.001. Error bars represent the standard error of the mean, *N* = 11 for each concentration. Veh: Artificial pond water only.

Having observed the effects of ethanol on stimulated stereotypical behaviour and locomotor activity in *L. variegatus*, we sought to determine the time‐dependent onset of ethanol action. Ethanol exerted inhibitory effects on the locomotor activity of *L. variegatus* at 250 mM and 500 mM after 2 min with movement reduced by 35.59% ± 6.45% and 35.10% ± 8.70%, respectively (*F*_(3.65,36.54)_ = 3.744, *p* < 0.05, Figure 3, *N* = 11). Hypokinesis was observed at 250 and 500 mM ethanol at all time points (*p* < 0.05, Figure 3, *N* = 11).

Inhibitory effects of ethanol on *L. variegatus* locomotion reached a steady‐state after 2 min of exposure to 250 and 500 mM ethanol, no significant differences were observed between the hypokinetic effects at 2 min compared with all other time points up to 10 min of exposure (*p* > 0.05, Figure 3, *N* = 11).

### 3.3 Ethanol absorption by *L. variegatus*

Having observed that 500 mM ethanol reduced the ability of tactile stimulation to elicit stereotypical movement and decreased locomotor activity behaviours of *L. variegatus* after 10 min of exposure (Figures 2 and 3), we sought to determine whether ethanol was producing these behavioural outcomes as a result of internal or external effects on the organism. Using a protocol adapted from Mitchell et al.,^3^ we found that 10 min of 500 mM ethanol exposure resulted in an ethanol concentration of 9.33 ± 1.09 mM/mg of *L. variegatus* whole tissue homogenate.

### 3.4 Acute Tolerance

**FIGURE 4:**
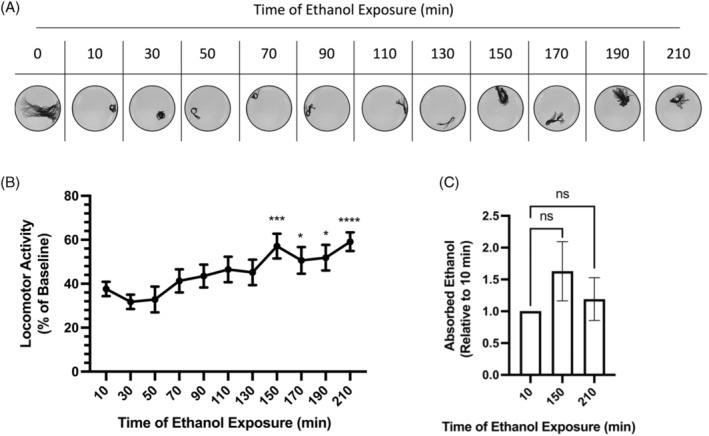
Acute ethanol tolerance in *Lumbriculus variegatus*. (A) Representative superimposed images analysed in ImageJ showing locomotor activity of *L. variegatus* exposed to 500 mM ethanol for up to 210 min. (B) Quantification of locomotor activity of *L. variegatus* exposed to 500 mM ethanol for 210 min with movement recorded at 20‐min intervals after 10 min of exposure. Data were expressed as a percentage of the area covered before ethanol exposure. Analyses compared baseline movements and movement after 10 min of exposure to ethanol using paired parametric two‐tailed *t*‐tests. All time points were significantly different (*p* < 0.0001) when compared with pre‐exposure locomotion., *N* = 24 for each time point. (C) Quantification of absorbed ethanol per μg of *L. variegatus* relative to ethanol absorbance after 10 min exposure to 500 mM ethanol. *N* = 4 with five *L. variegatus* per replicate, measured in duplicate. Analyses compared absorbed ethanol after 10 min of exposure to absorbed ethanol at 150 min and 210 min using one‐way ANOVA with Dunnett's post‐test. Error bars represent the standard error of the mean. ns, not significant, *p < 0.05, ***p < 0.001, ****p < 0.0001.

We observed that when *L. variegatus* was continuously exposed to 500 mM ethanol for 10 min, locomotor activity decreased to 37.65 ± 3.28% compared with pre‐exposure conditions (*t*_23_ = 19.04, *p* < 0.0001, Figure 4, *N* = 24). For all time points, exposure to 500 mM ethanol produced significant hypokinetic effects in *L. variegatus* compared with untreated controls (*p* < 0.0001, Figure 4, *N* = 24). However, locomotor activity during 500 mM ethanol exposure significantly increased over time. After 150 min of ethanol exposure, locomotion increased to 57.13 ± 5.65% of pre‐exposure conditions (*t*_23_ = 4.44, *p* = 0.0002, Figure 4, *N* = 24). All time points ≥ 150 min of ethanol exposure showed significant increases in locomotor ability compared with 10 min of ethanol exposure (*p* < 0.05, Figure 4, *N* = 24). Additionally, we observed that, even when behavioural depression is lessened, ethanol absorption by *L. variegatus* after 150 and 210 min of exposure were indistinguishable from the levels absorbed after exposure to 500 mM ethanol for 10 min (F_(1.60,3.71)_ = 1.766, *p* > 0.05, *N* = 4).

## 4 DISCUSSION

In this study, we observed that ethanol administered at doses similar to physiologically relevant concentrations in humans^2^ did not affect *L. variegatus* ability to respond to tactile stimulation during or after ethanol exposure (Figure 1A–D). Previous studies have shown that low‐dose ethanol can stimulate locomotion in *C. elegans* ^37^ and *Drosophila* ^38^ but we observed no stimulatory effect on *L. variegatus* locomotion (Figure 1E–G). We have previously showed that 10 min of exposure to drug compounds is sufficient to produce observable effects in *L. variegatus* ^31^, ^34^ and this time point has been used in previous invertebrate ethanol studies.^3^, ^38^

In *C. elegans* studies, high external concentrations of ethanol were used because it is believed that there is poor absorbance through the nematode cuticle,^39^ with the *C. elegans* cuticle presenting a barrier to the entry of other drugs.^3^ Because of this, we used ethanol concentrations similar to those used in previous *C. elegans* studies.^3^ We observed that exposure to 250–500 mM alcohol reduced the ability of tactile stimulation to elicit stereotypical movements of body reversal and helical swimming, and locomotor activity of *L. variegatus* (Figure 2) which mirrors findings in *C. elegans*.^3^ The inhibitory effects of ethanol were readily reversible after 10 min in artificial pond water, analogous to the response observed in *C. elegans*.^3^, ^6^

Next, we sought to determine the time‐dependent effects of ethanol‐induced hypokinesis of *L. variegatus*. We observed that 250 and 500 mM ethanol reached steady‐state levels after 2 min of exposure (Figure 3). This is a more rapid onset of action than findings in *C. elegans*, which reached steady‐state effects at 500 mM ethanol after 5 min.^3^ The route of entry of ethanol into *L. variegatus*, as with *C. elegans*, may be either directly across the cuticle and/or by ingestion through the mouth. The unlikelihood of such a rapid steady‐state by ingestion has previously been discussed^3^ and so equilibrium across the cuticle is the more likely route of entry into *L. variegatus*. The rapid onset of hypokinesis observed here may be because of the thinner cuticle observed in *L. variegatus* compared with *C. elegans*, as showed in transmission electron microscopy studies.^40^, ^41^

We measured ethanol absorption in *L. variegatus* using an established biochemical assay.^42^, ^43^ Because of the thin cuticle of *L. variegatus* in comparison to *C. elegans*, and high water solubility of ethanol, this may result in an underestimation of ethanol absorption in the animal because of internalised ethanol equilibrating with washing buffers, as previously proposed in similar studies of *C. elegans*.^3^

Finally, we showed that when *L. variegatus* are exposed to 500 mM ethanol, they showed a period of initial sensitivity followed by a recovery over time (Figure 4A,B). As the level of absorbed ethanol after 10 min of exposure was indistinguishable from levels after 150 and 210 min of ethanol exposure when behavioural depression was lessened (Figure 4C), this indicates the emergence of acute, or in session, tolerance.^8^, ^15^, ^21^ In this species, acute tolerance developed after 150 min of continuous ethanol exposure which is slower than studies in *C. elegans* which have showed acute tolerance within 30 min of exposure at concentrations equal to those used within this study.^8^

Furthermore, *L. variegatus* present some advantages over other invertebrate models used in ethanol studies. *L*. *variegatus* are larger in size (50–80 mm) compared with *C*. *elegans* (~1 mm) enabling individual organisms to be analysed without the requirement of microscopy equipment, while the thinner cuticle of *L. variegatus* compared with *C. elegans* reduces this as a barrier for ethanol entry. Additionally, fewer *L. variegatus* are required when studying the effects of ethanol; for example, measuring ethanol absorption may require hundreds of *C. elegans* ^3^, ^8^ while only five *L. variegatus* are required. Finally, in future studies, *L. variegatus* may provide additional insights for ethanol studies because of their longer lifespan^44^ compared with *C. elegans*.

## 5 CONCLUSION

In this study, we have used a combination of behavioural assays to define concentrations of ethanol that result in hypokinesis of *L. variegatus* while using established biochemical assays to quantify the absorption of ethanol. We show the effects of ethanol exposure on decreased response to tactile stimulation and depression of locomotory activity. Moreover, we observe the emergence of acute tolerance.

Our results indicate that the behavioural responses of *L. variegatus* are comparable to those of more extensively studied invertebrate models, such as *C. elegans* and *Drosophila*, and those seen in higher organisms. While genetic factors that mediate the response to ethanol have been characterised in *C. elegans* and *Drosophila* ^6^, ^8^, ^9^, ^12^, ^13^, ^22^, ^37^ there is limited genomic information on *L. variegatus* ^45^, ^46^, ^47^, ^48^ despite having a predicted genome size of larger than the domestic mouse at 2.64 Gbp.^49^ Future studies may show the utility of this novel model organism in the investigation of ethanol's genomic and molecular effects as well as pharmacology more broadly.

## AUTHOR CONTRIBUTIONS

**Aidan Seeley:** Conceptualisation, methodology, formal analysis, investigation, writing—original draft, visualisation, supervision, project administration, funding acquisition. **Romessa Mahmood:** Formal analysis, investigation, visualisation. **Caitlin Bellamy:** Formal analysis, investigation, visualisation. **Elis G. Roome:** investigation, visualisation. **Benjamin S. Williams:** Formal analysis, investigation, visualisation. **Nia A. Davies:** supervision, project administration, writing—review and editing. **Melisa J. Wallace:** supervision, project administration, writing—review and editing.

## FUNDING INFORMATION

This work is supported by a St David's Medical Foundation Seed‐Corn Grant.

## CONFLICT OF INTEREST STATEMENT

The authors declare no conflicts of interest.

## ETHICS STATEMENT

As invertebrates, *L. variegatus* are not covered under the Animal (Scientific Procedures) Act 1986 and, therefore, ethical approval was not required for the work presented.

## Data Availability

The data that support the findings of this study are available from the corresponding author upon reasonable request.

## References

[1] Jang GR , Harris RZ . Drug interactions involving ethanol and alcoholic beverages. Expert Opin Drug Metab Toxicol. 2007;3:719‐731. 17916057 10.1517/17425255.3.5.719

[2] Lee J , Jee C , McIntire SL . Ethanol preference in *C. Elegans* . Genes Brain Behav. 2009;8:578‐585. 19614755 10.1111/j.1601-183X.2009.00513.x PMC2880621

[3] Mitchell PH , Bull K , Glautier S , Hopper NA , Holden‐Dye L , O'Connor V . The concentration‐dependent effects of ethanol on Caenorhabditis elegans behaviour. Pharmacogenomics J. 2007;7:411‐417. 17325734 10.1038/sj.tpj.6500440

[4] Scholz H , Ramond J , Singh CM , Heberlein U . Functional ethanol tolerance in *Drosophila* . Neuron. 2000;28:261‐271. 11086999 10.1016/s0896-6273(00)00101-x

[5] Carta M , Mameli M , Valenzuela CF . Alcohol potently modulates climbing fiber→Purkinje neuron synapses: role of metabotropic glutamate receptors. J Neurosci. 2006;26:1906‐1912. 16481422 10.1523/JNEUROSCI.4430-05.2006 PMC6674936

[6] Kwon JY , Hong M , Choi MS , et al. Ethanol‐response genes and their regulation analyzed by a microarray and comparative genomic approach in the nematode Caenorhabditis elegans. Genomics. 2004;83:600‐614. 15028283 10.1016/j.ygeno.2003.10.008

[7] Yoshimura M , Tabakoff B . Ethanol's actions on cAMP‐mediated signaling in cells transfected with type VII adenylyl cyclase. Alcohol Clin Exp Res. 1999;23:1457‐1461. 10512310

[8] Davies AG , Bettinger JC , Thiele TR , Judy ME , McIntire SL . Natural variation in the npr‐1 gene modifies ethanol responses of wild strains of *C. Elegans* . Neuron. 2004;42:731‐743. 15182714 10.1016/j.neuron.2004.05.004

[9] Wen T , Parrish CA , Xu D , Wu Q , Shen P . Drosophila neuropeptide F and its receptor, NPFR1, define a signaling pathway that acutely modulates alcohol sensitivity. Proc Natl Acad Sci. 2005;102:2141‐2146. 15677721 10.1073/pnas.0406814102 PMC548536

[10] Ariwodola OJ , Weiner JL . Ethanol potentiation of GABAergic synaptic transmission may Be self‐limiting: role of presynaptic GABAB receptors. J Neurosci. 2004;24:10679‐10686. 15564584 10.1523/JNEUROSCI.1768-04.2004 PMC6730127

[11] Barry JA , Gawrisch K . Direct NMR evidence for ethanol binding to the lipid‐water Interface of phospholipid bilayers. Biochemistry. 1994;33:8082‐8088. 8025114 10.1021/bi00192a013

[12] Davies AG , Pierce‐Shimomura JT , Kim H , et al. A central role of the BK potassium channel in behavioral responses to ethanol in *C. Elegans* . Cell. 2003;115:655‐666. 14675531 10.1016/s0092-8674(03)00979-6

[13] Singh CM , Heberlein U . Genetic control of acute ethanol‐induced behaviors in *Drosophila* . Alcohol Clin Exp Res. 2000;24:1127‐1136. 10968649

[14] Davies AG , McIntire SL . Using *C. Elegans* to screen for targets of ethanol and behavior‐altering drugs. Biol Proced Online. 2004;6:113‐119. 15192754 10.1251/bpo79 PMC420456

[15] Dhawan R , Dusenbery DB , Williams PL . Comparison of lethality, reproduction, and behavior as toxicological endpoints in the nematode Caenorhabditis elegans. J Toxicol Environ Health A. 1999;58:451‐462. 10616193 10.1080/009841099157179

[16] Morgan PG , Sedensky MM . Mutations affecting sensitivity to ethanol in the nematode, *Caenorhabditis elegans* . Alcohol Clin Exp Res. 1995;19:1423‐1429. 8749805 10.1111/j.1530-0277.1995.tb01002.x

[17] Crabbe JC , Rigter H , Uijlen J , Strijbos C . Rapid development of tolerance to the hypothermic effect of ethanol in mice. J Pharmacol Exp Ther. 1979;208(1):128‐133. 759607

[18] Kalant H . Research on tolerance: what can we learn from history? Alcohol Clin Exp Res. 1998;22(1):67‐76. doi:10.1111/j.1530-0277.1998.tb03618.x 9514287

[19] LeBlanc AE , Kalant H , Gibbins RJ . Acute tolerance to ethanol in the rat. Psychopharmacologia. 1975;41(1):43‐46. doi:10.1007/BF00421304 1124268

[20] Pietrzykowski AZ , Treistman SN . The molecular basis of tolerance. Alcohol Res Health. 2008;31(4):298‐309. 23584007 PMC3860466

[21] Berger KH , Heberlein U , Moore MS . Rapid and chronic: two distinct forms of ethanol tolerance in drosophila. Alcohol Clin Exp Res. 2004;28:1469‐1480. 15597078 10.1097/01.alc.0000141817.15993.98

[22] Cowmeadow RB , Krishnan HR , Atkinson NS . The slowpoke gene is necessary for rapid ethanol tolerance in *drosophila* . Alcohol Clin Exp Res. 2005;29:1777‐1786. 16269907 10.1097/01.alc.0000183232.56788.62

[23] Larnerd C , Adhikari P , Valdez A , Toro AD , Wolf FW . Rapid and chronic ethanol tolerance are composed of distinct memory‐like states in *drosophila* . J Neurosci. 2023;43:2210‐2220. 36750369 10.1523/JNEUROSCI.1348-22.2023 PMC10039739

[24] Drewes CD . Helical swimming and body reversal behaviors in *Lumbriculus variegatus* (Annelida: Clitellata: Lumbriculidae). Aquatic Oligochaetes. Springer Netherlands; 1999:263‐269.

[25] Aikins DM , Mehler WT , Veilleux HD , Zhang Y , Goss GG . The acute and chronic effects of a sediment‐bound synthetic musk, galaxolide, on *Hyalella azteca*, *Chironomus dilutus*, and *Lumbriculus variegatus* . Arch Environ Contam Toxicol. 2023;84:227‐236. 36653626 10.1007/s00244-023-00978-3

[26] Colombo V , Pettigrove VJ , Hoffmann AA , Golding LA . Effects of *Lumbriculus variegatus* (Annelida, oligochaete) bioturbation on zinc sediment chemistry and toxicity to the epi‐benthic invertebrate *Chironomus tepperi* (Diptera: Chironomidae). Environ Pollut. 2016;216:198‐207. 27262133 10.1016/j.envpol.2016.05.063

[27] O'Gara BA , Bohannon VK , Teague MW , Smeaton MB . Copper‐induced changes in locomotor behaviors and neuronal physiology of the freshwater oligochaete, *Lumbriculus variegatus* . Aquat Toxicol. 2004;69:51‐66. 15210297 10.1016/j.aquatox.2004.04.006

[28] Sardo AM , Soares AMVM . Assessment of the effects of the pesticide imidacloprid on the behaviour of the aquatic oligochaete *Lumbriculus variegatus* . Arch Environ Contam Toxicol. 2010;58:648‐656. 20127481 10.1007/s00244-010-9470-0

[29] Silva CJM , Patrício Silva AL , Campos D , Soares AMVM , Pestana JLT , Gravato C . *Lumbriculus variegatus* (oligochaeta) exposed to polyethylene microplastics: biochemical, physiological and reproductive responses. Ecotoxicol Environ Saf. 2021;207:111375. 32987189 10.1016/j.ecoenv.2020.111375

[30] Vought V , Wang H‐S . Impact of common environmental chemicals bisphenol a and bisphenol S on the physiology of *Lumbriculus variegatus* . Environ Toxicol Pharmacol. 2018;60:225‐229. 29763883 10.1016/j.etap.2018.05.003

[31] Carriere JJ , Davies NA , Cunningham MR , Wallace MJ , Seeley A . Co‐created in vivo pharmacology practical classes using the novel organism Lumbriculus variegatus. Pharmacol Res Perspect. 2023;11:e01158. 38063050 10.1002/prp2.1158 PMC10704400

[32] Karlsson MV , Marshall S , Gouin T , Boxall ABA . Routes of uptake of diclofenac, fluoxetine, and triclosan into sediment‐dwelling worms. Environ Toxicol Chem. 2016;35:836‐842. 25892588 10.1002/etc.3020

[33] Nentwig G . Effects of pharmaceuticals on aquatic invertebrates. Part II: the antidepressant drug fluoxetine. Arch Environ Contam Toxicol. 2007;52:163‐170. 17160491 10.1007/s00244-005-7190-7

[34] Seeley A , Bellamy C , Davies NA , Wallace MJ . *Lumbriculus variegatus*: a novel organism for in vivo pharmacology education. Pharmacol Res Perspect. 2021;9:e00853. 34415088 10.1002/prp2.853 PMC8380063

[35] Matta SG , Balfour DJ , Benowitz NL , et al. Guidelines on nicotine dose selection for in vivo research. Psychopharmacology. 2007;190:269‐319. 16896961 10.1007/s00213-006-0441-0

[36] Bradford MM . A rapid and sensitive method for the quantitation of microgram quantities of protein utilizing the principle of protein‐dye binding. Anal Biochem. 1976;72:248‐254. 942051 10.1016/0003-2697(76)90527-3

[37] Johnson JR , Edwards MR , Davies H , et al. Ethanol stimulates locomotion via a Gαs‐signaling pathway in IL2 neurons in *Caenorhabditis elegans* . Genetics. 2017;207:1023‐1039. 28951527 10.1534/genetics.117.300119 PMC5676223

[38] Wolf FW , Rodan AR , Tsai LT‐Y , Heberlein U . High‐resolution analysis of ethanol‐induced locomotor stimulation in *Drosophila* . J Neurosci. 2002;22:11035‐11044. 12486199 10.1523/JNEUROSCI.22-24-11035.2002 PMC6758417

[39] Alaimo JT , Davis SJ , Song SS , et al. Ethanol metabolism and osmolarity modify behavioral responses to ethanol in *ssss* . Alcohol Clin Exp Res. 2012;36:1840‐1850. 22486589 10.1111/j.1530-0277.2012.01799.x PMC3396773

[40] Pakarinen K , Petersen EJ , Leppänen MT , Akkanen J , Kukkonen JVK . Adverse effects of fullerenes (nC60) spiked to sediments on *Lumbriculus variegatus* (Oligochaeta). Environ Pollut. 2011;159:3750‐3756. 21852027 10.1016/j.envpol.2011.07.014

[41] Peixoto CA , Kramer JM , de Souza W . Caenorhabditis elegans cuticle: a description of new elements of the fibrous layer. J Parasitol. 1997;83:368‐372. 9194814

[42] Lim YW , Meyer NP , Shah AS , Budde MD , Stemper BD , Olsen CM . Voluntary alcohol intake following blast exposure in a rat model of mild traumatic brain injury. PLoS One. 2015;10:e0125130. 25910266 10.1371/journal.pone.0125130 PMC4409117

[43] Patton MS , Heckman M , Kim C , Mu C , Mathur BN . Compulsive alcohol consumption is regulated by dorsal striatum fast‐spiking interneurons. Neuropsychopharmacology. 2021;46:351‐359. 32663841 10.1038/s41386-020-0766-0 PMC7852608

[44] Ji CW , Park Y‐S , Cui Y , Wang H , Kwak I‐S , Chon T‐S . Analyzing the response behavior of *Lumbriculus variegatus* (Oligochaeta: Lumbriculidae) to different concentrations of copper sulfate based on line body shape detection and a recurrent self‐organizing map. Int J Environ Res Public Health. 2020;17(8):2627. doi:10.3390/ijerph17082627 32290455 PMC7215344

[45] Anderson FE , Williams BW , Horn KM , et al. Phylogenomic analyses of Crassiclitellata support major northern and southern hemisphere clades and a Pangaean origin for earthworms. BMC Evol Biol. 2017;17:123. 28558722 10.1186/s12862-017-0973-4 PMC5450073

[46] Gustafsson DR , Price DA , Erséus C . Genetic variation in the popular lab worm *Lumbriculus variegatus* (Annelida: Clitellata: Lumbriculidae) reveals cryptic speciation. Mol Phylogenet Evol. 2009;51:182‐189. 19141324 10.1016/j.ympev.2008.12.016

[47] Phillips AJ , Dornburg A , Zapfe KL , et al. Phylogenomic analysis of a putative missing link Sparks reinterpretation of leech evolution. Genome Biol Evol. 2019;11:3082‐3093. 31214691 10.1093/gbe/evz120 PMC6598468

[48] Tellez‐Garcia AA , Álvarez‐Martínez R , López‐Martínez JM , Arellano‐Carbajal F . Transcriptome analysis during early regeneration of *Lumbriculus variegatus* . Gene Reports. 2021;23:101050.

[49] Tweeten KA , Morris SJ . Flow cytometry analysis of DNA ploidy levels and protein profiles distinguish between populations of *Lumbriculus* (Annelida: Clitellata). Invertebrate Biology. 2016;135:385‐399.

